# Effects of neuromuscular training on physical and tennis-specific performance in youth and young adult male tennis players: A systematic review and meta-analysis

**DOI:** 10.1371/journal.pone.0355297

**Published:** 2026-08-05

**Authors:** Pingping Xie, Jinfa Gu, Jie Huang

**Affiliations:** 1 Office of Academic Affairs, Guilin University of Technology, Guilin, China; 2 Exercise & Sports Science Programme, School of Health Sciences, Universiti Sains Malaysia, Kubang Kerian, Kelantan, Malaysia; 3 School of Physical Education, Yancheng Kindergarten Teachers College, Yancheng, Jiangsu, China; Ondokuz Mayıs Üniversitesi: Ondokuz Mayis Universitesi, TÜRKIYE

## Abstract

**Background:**

Neuromuscular training (NMT) is used to improve physical fitness and tennis-specific performance, but broad outcome classifications, mixed populations, and insufficient separation of accuracy and sprint outcomes have limited previous reviews. This systematic review and meta-analysis evaluated the effects of NMT in male tennis players, particularly youth and young adults.

**Methods:**

Five databases were searched from inception to 1 January 2026; the protocol was registered in PROSPERO (CRD420261337037). Eligible studies were randomized controlled trials (RCTs) or non-RCTs involving healthy male tennis players and comparing structured NMT with control or alternative training conditions. Outcomes included stroke velocity, shot placement accuracy, serve accuracy, sprint performance, countermovement jump (CMJ), and agility time. Risk of bias was assessed using RoB 2 and ROBINS-I, certainty of evidence was evaluated with GRADE, and random-effects meta-analyses were performed using Hedges’ g.

**Results:**

Eighteen studies involving 670 participants were included, of which 16 were RCTs and two were non-randomized controlled studies. Compared with control conditions, pooled estimates favored the intervention conditions for stroke velocity (g = 0.95), sprint performance (g = −0.58), CMJ (g = 0.73), agility time (g = −0.89), and serve accuracy (g = 0.47). No significant pooled effect was observed for shot placement accuracy (g = −0.41). For sprint performance, the pooled estimate favored the intervention conditions for the 10 m sprint but not for the 20 m sprint. GRADE certainty was low for stroke velocity and sprint performance and very low for shot placement accuracy, serve accuracy, CMJ, and agility time.

**Conclusions:**

Low-certainty evidence suggests favorable pooled effects on stroke velocity and short-distance sprint performance when the included interventions were considered collectively. Although pooled analyses favored the intervention conditions for CMJ, agility time, and serve accuracy, the certainty of evidence for these outcomes was very low, and accuracy-related outcomes remained inconsistent. These pooled findings do not establish the effectiveness of any specific training modality. Further rigorously designed and well-reported RCTs incorporating long-term follow-up are warranted.

## 1. Introduction

Tennis is a high-intensity, intermittent sport requiring repeated acceleration, deceleration, changes of direction, and stroke execution [1]. Match-analysis evidence also shows that change-of-direction actions occur frequently during professional tennis matchplay, underscoring the importance of neuromuscular qualities for tennis performance [2]. These demands require high levels of speed, explosive power, agility, and sport-specific technical control, and physical fitness is considered an important determinant of match performance and competitive level [3]. Higher stroke velocity during serves and offensive strokes is also considered an important characteristic of tennis-specific performance [4]. Accordingly, identifying effective training strategies to improve physical fitness and tennis-specific performance remains an important focus of tennis research.

Despite the growing number of systematic reviews and meta-analyses, several important limitations remain [5]. First, previous reviews have often used broad outcome classifications, with limited differentiation among peak serve velocity, mean serve velocity, and groundstroke velocity [3]. This limited differentiation may reduce the practical value of review findings for tennis-specific training. Second, previous reviews have often included mixed-sex samples or participants across wide age ranges [3,5]. Sex-related differences in strength, muscle characteristics, and neuromuscular adaptations, together with maturation-related differences in youth athletes, may influence training responses [6–8]. Youth and young adult male players may also differ in maturation status, strength base, and movement stability and control [6,7]. Pooling these groups may therefore complicate interpretation of the pooled estimates [8].

Evidence focused specifically on male tennis players, particularly youth and young adults, remains limited [3,9]. Although a recent systematic review and meta-analysis synthesized NMT interventions in tennis players, uncertainty remains regarding evidence in male players and the interpretation of specific tennis-performance outcomes [5]. Accuracy-related evidence also remains unclear because existing studies have focused primarily on velocity- and fitness-related outcomes, and it remains uncertain whether changes in accuracy accompany improvements in velocity [10–12]. Serve accuracy and shot placement accuracy have also not consistently been examined separately [4].

In response to these limitations, the present review aimed to synthesize NMT-related evidence in male tennis players, particularly youth and young adults. Rather than replacing previous broader reviews, it addressed a more specific question by restricting the population to male players and separately examining stroke-velocity subtypes, 10 m and 20 m sprint performance, serve accuracy, shot placement accuracy, CMJ, and agility time. Recent tennis-specific training studies and scoping evidence further support distinguishing physical performance outcomes from tennis-specific accuracy outcomes [13–15].

Therefore, the review aimed to clarify which domains showed relatively consistent findings and which remained uncertain, while also exploring age-related subgroup patterns where data allowed.

## 2. Materials and methods

### 2.1 Registration and reporting standards

This systematic review and meta-analysis were conducted and reported in accordance with the Preferred Reporting Items for Systematic Reviews and Meta-Analyses (PRISMA) 2020 statement and the methodological recommendations of the Cochrane Handbook for Systematic Reviews of Interventions [16]. The review protocol was registered in the International Prospective Register of Systematic Reviews (PROSPERO) (CRD420261337037).

### 2.2 Search strategy

Two reviewers independently conducted a systematic search of PubMed, Web of Science, Scopus, Cochrane Library and Embase from inception to 1 January 2026. The search strategy combined controlled vocabulary, such as MeSH and Emtree terms, with free-text keywords related to tennis and NMT, including plyometric training, resistance training, core training, and other NMT modalities. Boolean operators (AND/OR) were applied as appropriate. No sex restriction was applied during the database search; eligibility by sex was determined during study screening. To reduce the risk of missing relevant studies, the reference lists of all included articles and relevant reviews were also screened manually. Only full-text articles published in English were considered eligible, with no restriction on publication year. The full search strategies for all databases are provided in Supplementary S1 Table.

### 2.3 Study selection and screening process

All records retrieved from the electronic searches were imported into reference management software, and duplicate records were removed. Study selection was conducted in two stages according to the prespecified eligibility criteria. First, titles and abstracts were screened independently by two reviewers. Second, the full texts of potentially eligible studies were assessed for inclusion. Any disagreements were resolved through discussion; if consensus could not be reached, a third reviewer was consulted.

### 2.4 Eligibility criteria

#### 2.4.1 Inclusion criteria.

**Participants**: Healthy male tennis players. Only studies with exclusively male samples were included.

**Intervention:** Structured exercise interventions implemented alongside routine tennis practice or as a partial substitute for it and primarily targeting one or more tennis-relevant physical or motor capacities, including force production, power, coordination, trunk or postural control, agility, footwork, or sport-specific movement control. For the purposes of this review, NMT was used as an operational umbrella term for biomechanically distinct modalities, including resistance, plyometric, isometric, flywheel, core, medicine-ball, agility, footwork, functional, and multicomponent training; these modalities were not considered equivalent or interchangeable interventions.

**Comparators:** Usual training, routine tennis practice, passive control, or alternative non-NMT training.

**Outcomes:** At least one extractable outcome related to tennis-specific or physical performance, including stroke velocity, shot placement accuracy, serve accuracy, sprint performance, countermovement jump (CMJ), or agility time.

**Study design:** Randomized controlled trials (RCTs) and non-RCTs with sufficient data for quantitative synthesis.

#### 2.4.2 Exclusion criteria.

Studies were excluded if they did not involve healthy male tennis players, included female participants or mixed-sex samples without separate male data, involved injured, clinical, or other special populations, did not examine NMT as the main intervention or as a clearly defined part of the training program, lacked an eligible control/comparator group, or did not report extractable data for at least one relevant outcome.

### 2.5 Data extraction and data handling

Two reviewers independently extracted data using a predesigned standardized form. The extracted information included the first author and publication year, study design, sample size, participant characteristics, and detailed information on the intervention and control conditions, including training type, intervention duration, training frequency, and session characteristics. Data for the prespecified outcomes were also extracted. When outcome data were missing, incomplete, or unclear, extractable information was obtained from the published reports, tables, figures, and supplementary materials where possible. Study authors were not contacted for additional data. Duplicate or overlapping reports were checked by comparing study characteristics.

According to the predefined analytical framework, outcomes were categorized as stroke velocity, shot placement accuracy, serve accuracy, sprint performance, CMJ, and agility time. For outcomes eligible for further stratified analyses, stroke velocity was classified by specific subtype, and sprint performance was classified according to testing distance. Age-related information was also extracted to support subgroup analyses comparing youth and young adult male players. Included interventions were also classified descriptively according to their dominant training component and reported intervention content. A study-level descriptive classification of the included interventions is provided in Supplementary S3 Table.

Any disagreements arising during data extraction were resolved through discussion between the two reviewers and, when necessary, adjudicated by a third reviewer. For continuous outcomes, post-intervention means and standard deviations were preferentially extracted for meta-analysis because most studies did not report change scores consistently. Baseline comparability between intervention and control groups was additionally examined for non-randomized studies using available baseline means and SDs. Hedges’ g was used as the pooled effect size for most outcomes to reduce small-sample bias and improve comparability across different outcome measures. In addition, to enhance interpretability in the original units, raw data for 10 m and 20 m sprint times were also extracted and analyzed using mean differences (MDs). When multiple related indicators were reported within a single study, they were categorized according to the predefined outcome framework, and efforts were made to avoid double-counting the same participant sample within a single pooled analysis.

### 2.6 Risk of bias and methodological quality assessment

Risk of bias was assessed according to the study design. RCTs were evaluated using the Cochrane Risk of Bias 2 (RoB 2) tool [17], whereas non-randomized controlled studies were assessed using the Risk of Bias in Non-randomized Studies of Interventions (ROBINS-I) tool [18]. Two reviewers independently conducted the assessments, with disagreements resolved through discussion or, if required, adjudication by a third reviewer. The results were summarized graphically.

### 2.7 Certainty of evidence (GRADE)

The certainty of evidence for each outcome was assessed using the Grading of Recommendations Assessment, Development and Evaluation (GRADE) framework [19]. Evidence was rated as high, moderate, low, or very low across the domains of risk of bias, inconsistency, indirectness, imprecision, and publication bias. Given that both RCTs and non-randomized controlled studies were included, certainty judgments took study design into account. Two reviewers independently performed the GRADE assessment, and disagreements were resolved through discussion and consensus.

### 2.8 Statistical analysis

All meta-analyses were performed using random-effects models [20,21]. Continuous outcomes were pooled as standardized mean differences (SMDs) with 95% confidence intervals (CIs), estimated using Hedges’ g to correct for small-sample bias [22]. Effect directions were aligned before pooling to ensure consistent interpretation within each outcome. Specifically, for sprint time and agility time, lower post-intervention values indicate better performance; therefore, negative effect sizes indicate a favorable intervention effect. For shot placement accuracy, where lower values reflect greater accuracy, negative effect sizes also indicate improvement. By contrast, for stroke velocity, CMJ height, and serve accuracy, higher values indicate better performance, and positive effect sizes indicate a favorable intervention effect. Serve accuracy and shot placement accuracy were treated as separate accuracy-related outcomes because the underlying testing protocols and scoring systems differed across studies.

Statistical heterogeneity was assessed using the I^2^ statistic. I^2^ values of approximately 25%, 50%, and 75% were considered to indicate low, moderate, and high heterogeneity, respectively [23].

Where sufficient data were available, subgroup analyses were conducted according to outcome subtype and age group. Age subgroup analyses were performed only for outcomes with sufficient numbers of studies in both age categories to allow meaningful comparison. Because maturation-related indicators were rarely reported, age subgroup analyses were exploratory and based on chronological age only. Youth and young adult subgroups were defined as 9–17 years and 18–30 years, respectively, according to the reported mean age or age range of each study. Hedges’ g was retained as the main pooled effect metric across outcomes. For sprint outcomes, additional analyses using mean differences (MDs) were performed separately for 10 m and 20 m sprint times to facilitate interpretation in the original unit of seconds.

Sensitivity analyses were conducted using a leave-one-out approach. Additional sensitivity analyses were performed by comparing fixed-effect and random-effects models and by excluding non-randomized studies.

Publication bias was assessed visually using funnel plots and statistically using Egger’s regression test and Begg’s rank correlation test. The trim-and-fill method was additionally applied to explore the potential influence of publication bias on the pooled estimates [24–26]. For outcomes with fewer than 10 studies or effect sizes, publication-bias assessments were considered exploratory and were not interpreted as definitive evidence of the presence or absence of publication bias. A two-sided p value < 0.05 was considered statistically significant. All statistical analyses were performed using R via RStudio.

## 3. Results

### 3.1 Study selection

**Fig 1:**
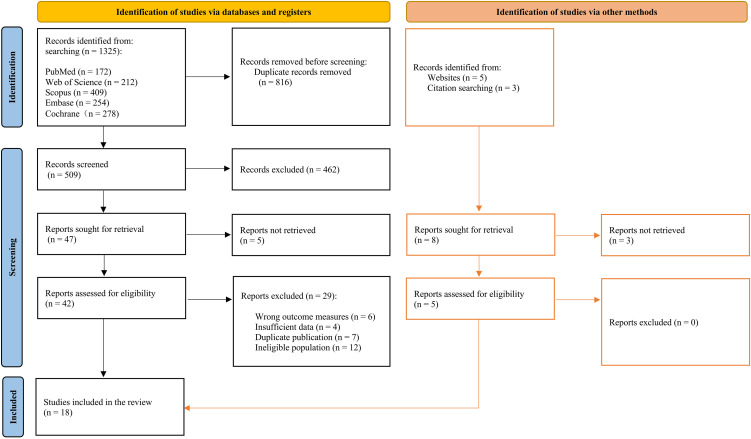
PRISMA flow diagram illustrating the study selection process for the systematic review and meta-analysis.

A total of 1,325 records were identified through database searching (Fig 1). After removal of 816 duplicates, 509 records were screened, and 462 were excluded. Full-text reports were sought for 47 records; 5 could not be retrieved, leaving 42 reports for eligibility assessment. An additional 8 records were identified through other methods, of which 3 could not be retrieved, and 5 were assessed for eligibility. After full-text review, 29 reports were excluded because of wrong outcome measures (n = 6), insufficient data (n = 4), duplicate publication (n = 7), or ineligible population (n = 12). In total, 18 studies were included in the review.

### 3.2 Study Characteristics

A total of 18 studies involving 670 participants were included in this review, of which 16 were RCTs and two were non-randomized controlled studies. Sample sizes ranged from 12 to 100, and the study populations consisted mainly of youth tennis players, with some studies also involving young adults [27–44]. Reported mean ages ranged from 9.6 to 26.9 years, although several studies reported age ranges rather than means. Information on Tanner stage, biological maturation, training age, and competitive level was rarely or inconsistently reported across the included studies.

Intervention duration ranged from 6 to 12 weeks, with 8-week programs being the most common. Training frequency was typically 2–3 sessions per week, with the overall range extending from 1–2 to 4 sessions per week. The intervention protocols were heterogeneous and included resistance and isometric strength training, plyometric and explosive-strength training, flywheel training, core and medicine-ball training, agility and footwork drills, functional training, and multicomponent programs. The prespecified outcomes covered both tennis-specific and general physical performance and included stroke velocity, shot placement accuracy, serve accuracy, sprint performance, CMJ, and agility time. The characteristics of the included studies are summarized in Table 1, and the descriptive classification of intervention types is provided in Supplementary S3 Table.

**Table 1:** Characteristics of included studies.

Study Citation	Sample (N)	Age (years)	Intervention Protocol	Main Intervention Content	Outcome Measures
Baiget et al. (2022)	16	15.6 ± 1.1	6 weeks, 2–3 sessions/wk	Joint-specific isometric strength training (SHIR & SHF)	Serve Velocity
Behringer et al. (2013)	33	15.03 ± 1.64	8 weeks, 2 sessions/wk	Traditional resistance vs. Plyometric training	Serve Velocity; Service Precision
Canós et al. (2022)	24	15.5 ± 1.2	8 weeks, 2 sessions/wk	Machine-based (MNMT) vs. Flywheel (FNMT) training	Serve Velocity; CMJ; Agility
Fernandez-Fernandez et al. (2013)	30	14.2 ± 0.5	6 weeks, 3 sessions/wk	Core strength, elastic resistance, and medicine ball	Serve Velocity; Serve Accuracy
Fernandez-Fernandez et al. (2015)	16	16.9 ± 0.5	8 weeks, 2 sessions/wk	Combined explosive strength and repeated sprint training	Sprint (10 m, 20 m); CMJ
Fernandez-Fernandez et al. (2016)	51	12.5 ± 0.3	8 weeks, 2 sessions/wk	Upper- and lower-body integrated plyometric training	Serve Velocity; Serve Accuracy; Sprint; CMJ; Agility
Gamlath & Thotawaththa (2023)	16	16.4 ± 0.5	6 weeks, 2–3 sessions/wk	Lower-extremity plyometric activities	Sprint (10 m); Agility; CMJ
Genevois et al. (2013)	44	26.9 ± 7.5	6 weeks, 2 sessions/wk	Handled medicine ball (HMB) vs. Overweight racket (OWR)	Forehand Velocity; Shot Accuracy
Kong & Xu (2023)	100	21	12 weeks	Progressive medicine ball strength training	Serve Velocity; Upper-limb Explosive Power
Mainer-Pardos et al. (2024)	12	13.4 ± 0.36	10 weeks, 2 sessions/wk	Neuromuscular training (speed, strength, throws, agility, jumps, and coordination)	Sprint (20 m); CMJ; Agility
Paul et al. (2011)	30	18–24	8 weeks, 4 sessions/wk	Sport-specific agility and footwork drills	Serve Accuracy; Agility
Rathore (2016)	60	18–23	8 weeks, 3 sessions/wk	Plyometric training vs. Traditional resistance training	Agility
Salonikidis & Zafeiridis (2008)	64	21.1 ± 1.3	9 weeks, 3 sessions/wk	Plyometric vs. Tennis-specific drills vs. Combined	CMJ; Agility; Speed
Sinkovic et al. (2023)	35	12.14 ± 1.3	6 weeks, 2 sessions/wk	Lower-limb plyometric training	Sprint (10 m, 20 m); CMJ; Agility
Wang & Xu (2025)	47	15.6 ± 0.9	8 weeks, 1–2 sessions/wk	Upper-limb plyometric training (ULPT)	Serve Velocity; ISRT/ESRT
Yildiz et al. (2019)	28	9.6 ± 0.7	8 weeks, 3 sessions/wk	Functional training (FT) vs. Traditional training	Sprint (10 m); CMJ; Agility
Ölçücü et al. (2013)	40	20–25	8 weeks, 3 sessions/wk	Progressive plyometric training	Serve Velocity; Hitting Percentage
Ziagkas et al. (2019)	24	20.9 ± 0.66	8 weeks, 2 sessions/wk	Plyometric training program integrated into tennis practice	Agility

*N*, sample size; sessions/wk, sessions per week; CMJ, countermovement jump; SHIR, shoulder internal rotation; SHF, shoulder flexion; MNMT, machine-based neuromuscular training; FNMT, flywheel neuromuscular training; HMB, handled medicine ball; OWR, overweight racket; ULPT, upper-limb plyometric training; FT, functional training; ISRT, internal shoulder rotation torque; ESRT, external shoulder rotation torque.

### 3.3 Risk of Bias Results

**Fig 2:**
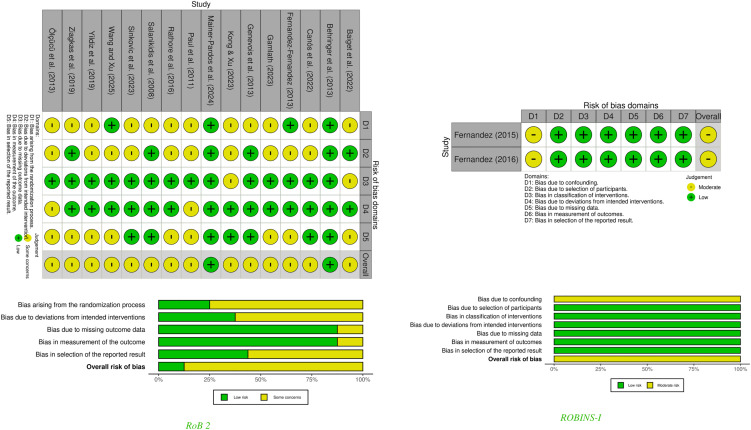
Risk-of-bias assessment of included studies using RoB 2 for RCTs and ROBINS-I for non-randomized controlled studies.

Risk of bias in the RCTs was assessed using the Cochrane RoB 2 tool (Fig 2). Of the 16 RCTs, 2 (12.5%) were judged to be at low risk of bias overall, whereas 14 (87.5%) raised some concerns; no trial was classified as high risk. Some concerns most frequently arose from the randomization process (D1; 12/16, 75.0%), followed by deviations from intended interventions (D2; 10/16, 62.5%) and selection of the reported result (D5; 9/16, 56.3%). By contrast, missing outcome data (D3) and measurement of the outcome (D4) were generally judged to be at low risk (14/16, 87.5% for both domains).

Risk of bias in the non-randomized studies was assessed using ROBINS-I. Both non-randomized studies were judged to be at moderate overall risk of bias, mainly due to bias arising from confounding, whereas the remaining domains were rated as low risk. Baseline comparability of the two non-randomized studies is summarized in Supplementary S2 Table.

### 3.4 Overall effects

Compared with control conditions, the pooled effects favored the intervention for stroke velocity (k = 12 effect sizes, total N = 369; Hedges’ g = 0.95, 95% CI 0.68 to 1.22; *p* < 0.001; I² = 23%), sprint performance (k = 10, total N = 260; g = −0.58, 95% CI −0.87 to −0.28; *p* = 0.0001; I² = 19%), CMJ (k = 10, total N = 214; g = 0.73, 95% CI 0.34 to 1.13; *p* = 0.0003; I² = 38%), agility time (k = 11, total N = 272; g = −0.89, 95% CI −1.22 to −0.56; *p* < 0.001; I² = 32%), and serve accuracy (scores/counts) (k = 5, total N = 141; g = 0.47, 95% CI 0.11 to 0.84; *p* = 0.01; I² = 13%).

**Fig 3:**
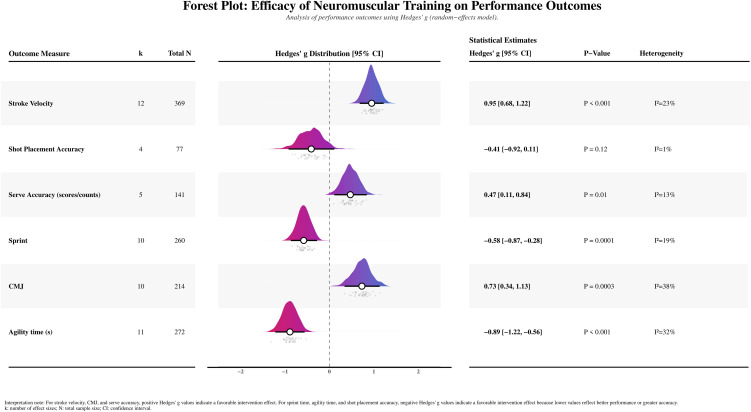
Forest plot of the effects of NMT-related interventions on performance outcomes.

No significant pooled effect was observed for shot placement accuracy (k = 4, total N = 77; g = −0.41, 95% CI −0.92 to 0.11; *p* = 0.12; I² = 1%). Although I² indicated low-to-moderate statistical heterogeneity, clinical heterogeneity remained across interventions, participants, and testing protocols. Accordingly, the pooled estimates should be interpreted as average outcome-level effects across heterogeneous NMT-related interventions rather than as modality-specific effects. The corresponding forest plot is shown in Fig 3, with detailed study-level forest plots provided in Supplementary S1 Fig.

### 3.5 Additional analyses

#### 3.5.1 Subgroup analyses of stroke velocity and sprint performance.

**Fig 4:**
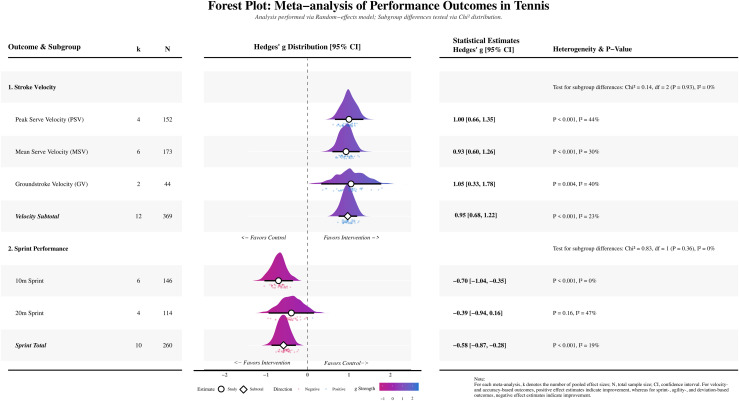
Subgroup analyses of stroke velocity and sprint performance.

Further subgroup analyses showed that the pooled effects favored the intervention across all stroke velocity subgroups, including peak serve velocity (k = 4, N = 152; Hedges’ g = 1.00, 95% CI 0.66 to 1.35; *p* < 0.001; I² = 44%), mean serve velocity (k = 6, N = 173; g = 0.93, 95% CI 0.60 to 1.26; *p* < 0.001; I² = 30%), and groundstroke velocity (k = 2, N = 44; g = 1.05, 95% CI 0.33 to 1.78; *p* = 0.004; I² = 40%). No significant subgroup differences were observed for stroke velocity (Chi² = 0.14, df = 2; *p* = 0.93; I² = 0%). For sprint performance, a significant pooled effect was observed for the 10 m sprint (k = 6, N = 146; g = −0.70, 95% CI −1.04 to −0.35; *p* < 0.001; I² = 0%), whereas no significant pooled effect was found for the 20 m sprint (k = 4, N = 114; g = −0.39, 95% CI −0.94 to 0.16; *p* = 0.16; I² = 47%). No significant subgroup differences were detected for sprint performance (Chi² = 0.83, df = 1; *p* = 0.36; I² = 0%). Subgroup analyses of stroke velocity and sprint performance are presented in Fig 4.

#### 3.5.2 Separate MD-based analyses of 10 m and 20 m sprint time.

**Fig 5:**
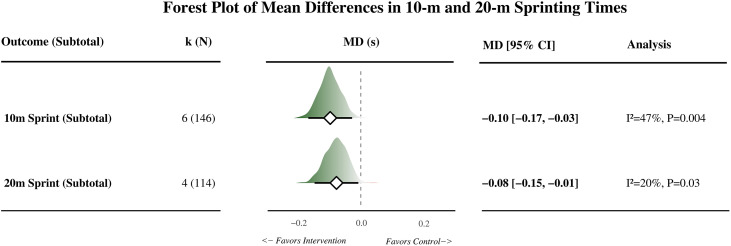
Separate MD-based analyses of 10-m and 20-m sprint time.

To facilitate interpretation in original units, separate analyses using MD showed significant reductions in both 10 m sprint time (k = 6, N = 146; MD = −0.10 s, 95% CI −0.17 to −0.03; *p* = 0.004; I² = 47%) and 20 m sprint time (k = 4, N = 114; MD = −0.08 s, 95% CI −0.15 to −0.01; *p* = 0.03; I² = 20%). Notably, this pattern differed from the SMD-based subgroup analysis, in which the pooled effect for the 20 m sprint did not reach statistical significance. Separate MD-based analyses of 10 m and 20 m sprint time are presented in Fig 5.

### 3.6 Subgroup analyses by age group

**Fig 6:**
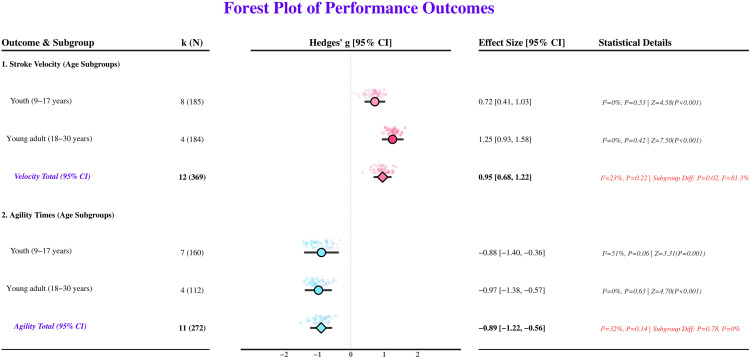
Subgroup analyses of stroke velocity and agility time by age group (youth vs. young adults).

Age subgroup analyses were exploratory and were limited to outcomes with sufficient numbers of studies in both age categories. For stroke velocity, significant pooled effects were observed in both the youth subgroup (9–17 years) (k = 8, N = 185; Hedges’ g = 0.72, 95% CI 0.41 to 1.03; *p* < 0.001; I² = 0%) and the young adult subgroup (18–30 years) (k = 4, N = 184; g = 1.25, 95% CI 0.93 to 1.58; *p* < 0.001; I² = 0%), with evidence of a between-subgroup difference (*p* = 0.02; I² = 81.3%). For agility time, significant pooled effects were also observed in both the youth subgroup (9–17 years) (k = 7, N = 160; g = −0.88, 95% CI −1.40 to −0.36; *p = 0.001*; I² = 51%) and the young adult subgroup (18–30 years) (k = 4, N = 112; g = −0.97, 95% CI −1.38 to −0.57; *p* < 0.001; I² = 0%); however, the between-subgroup difference was not significant (*p* = 0.78; I² = 0%). Subgroup analyses by age group are presented in Fig 6.

### 3.7 Sensitivity analysis

Sensitivity analyses using leave-one-out procedures and fixed-effect versus random-effects models yielded broadly consistent pooled estimates across outcomes, with no single effect size materially influencing the overall pattern of findings. Excluding the two non-randomized studies did not materially alter the direction of the pooled effects for any outcome. The statistical significance of most outcomes was unchanged, although the serve accuracy estimate no longer reached statistical significance at the conventional 0.05 level after the non-randomized studies were excluded. A targeted sensitivity analysis excluding the CMJ comparison with potential baseline imbalance in Fernandez-Fernandez et al. (2015) did not materially alter the result, with the pooled effect remaining significant (g = 0.63). The numerical results of sensitivity analyses are summarized in Supplementary S4 Table.

### 3.8 Assessment of publication bias

**Fig 7:**
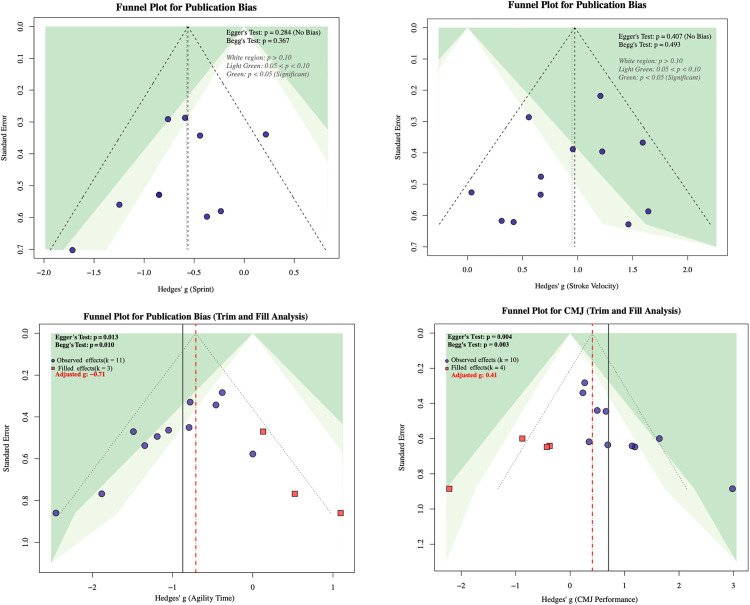
Funnel plots assessing publication bias.

Publication-bias assessments were interpreted cautiously, particularly for outcomes with fewer than 10 studies or effect sizes. Visual inspection of the funnel plots, together with Egger’s and Begg’s tests, suggested no clear evidence of small-study effects for stroke velocity (Egger’s p = 0.407; Begg’s p = 0.493) or sprint performance (Egger’s p = 0.284; Begg’s p = 0.367). In contrast, potential funnel-plot asymmetry was indicated for CMJ (Egger’s p = 0.004; Begg’s p = 0.003) and agility time (Egger’s p = 0.013; Begg’s p = 0.010). For CMJ, trim-and-fill analysis imputed four potentially missing effects and adjusted the pooled estimate to Hedges’ g = 0.41, while the direction of effect remained positive. For agility time, trim-and-fill analysis imputed three potentially missing effects and adjusted the pooled estimate to Hedges’ g = −0.71, with the direction of effect remaining unchanged. These findings suggest that small-study effects cannot be ruled out for CMJ and agility time, and the corresponding pooled estimates should therefore be interpreted cautiously (see Fig 7).

### 3.9 Certainty of evidence (GRADE)

The certainty of evidence for the main outcomes is presented in Table 2. Evidence was rated as low for stroke velocity and sprint performance and very low for shot placement accuracy, serve accuracy, CMJ, and agility time. Stroke velocity and sprint performance were downgraded mainly because of very serious risk of bias. Shot placement accuracy was downgraded because of very serious risk of bias and very serious imprecision, whereas serve accuracy was downgraded because of very serious risk of bias and serious imprecision. CMJ and agility time were downgraded because of very serious risk of bias and publication-bias concerns. Overall, confidence in the pooled estimates ranged from limited to very limited across outcomes.

**Table 2:** Certainty of evidence (GRADE framework) for main outcomes.

Outcomes	No. of participants and effect sizes	Pooled estimate, Hedges’ g [95% CI]	Risk of bias	Inconsistency	Indirectness	Imprecision	Publication bias	Certainty of evidence (GRADE)
Stroke velocity	N = 369;k = 12	0.95 [0.68, 1.22]	Very serious^a^	Not serious^b^	Not serious	Not serious	Not serious^e^	⊕⊕◯◯Low
Shot placement accuracy	N = 77;k = 4	−0.41 [−0.92, 0.11]	Very serious^a^	Not serious^b^	Not serious	Very serious^c^	Not serious^e^	⊕◯◯◯Very low
Serve accuracy	N = 141;k = 5	0.47 [0.11, 0.84]	Very serious^a^	Not serious^b^	Not serious	Serious^d^	Not serious^e^	⊕◯◯◯Very low
Sprint performance	N = 260;k = 10	−0.58 [−0.87, −0.28]	Very serious^a^	Not serious^b^	Not serious	Not serious	Not serious^e^	⊕⊕◯◯Low
Countermovement jump	N = 214;k = 10	0.73 [0.34, 1.13]	Very serious^a^	Not serious^b^	Not serious	Not serious	Serious^f^	⊕◯◯◯Very low
Agility time	N = 272;k = 11	−0.89 [−1.22, −0.56]	Very serious^a^	Not serious^b^	Not serious	Not serious	Serious^f^	⊕◯◯◯Very low

GRADE, Grading of Recommendations Assessment, Development and Evaluation; CI, confidence interval; CMJ, countermovement jump; N, total sample size; k, number of pooled effect sizes.

a. Downgraded for very serious risk of bias because most randomized studies had some concerns; for outcomes that also included non-randomized evidence, those studies were judged to have at least moderate risk of bias.

b. Not downgraded for inconsistency because statistical heterogeneity was generally low to moderate.

c. Downgraded for very serious imprecision because of the small sample size and confidence interval crossing the null effect.

d. Downgraded for serious imprecision because of the limited number of studies/participants and reduced robustness in sensitivity analysis.

e. Publication bias was not downgraded for these outcomes. For stroke velocity and sprint performance, formal tests did not suggest clear small-study effects; for shot placement accuracy and serve accuracy, formal assessment was limited because fewer than 10 effect sizes contributed to the pooled estimates.

f. Downgraded for publication-bias concerns because funnel-plot asymmetry and small-study effects were suggested by Egger’s and Begg’s tests, and trim-and-fill analyses attenuated the pooled effects.

## 4. Discussion

### 4.1 Main findings

This study systematically evaluated the effects of NMT interventions on physical fitness and tennis-specific performance in male tennis players, particularly youth and young adults. Overall, the pooled estimates favored the intervention conditions for stroke velocity, sprint performance, CMJ, agility time, and serve accuracy, whereas no statistically significant pooled effect was observed for shot placement accuracy. Further analyses indicated that the direction of effect was consistent across stroke-velocity subtypes. For sprint performance, the improvement signal appeared more consistent for the 10 m sprint than for the 20 m sprint, although this difference did not reach statistical significance. Age subgroup analyses showed a larger pooled effect for stroke velocity in young adults than in youth; however, this age-related difference was not consistently observed across all outcomes. Although sensitivity analyses generally supported the direction of the findings, GRADE assessments indicated that the certainty of evidence was low or very low for all main outcomes. Therefore, the present findings should be interpreted as providing cautious supportive evidence rather than conclusions confirmed by high-certainty evidence. Recent tennis-specific studies have also examined a range of physical and technical performance outcomes [13–15].

### 4.2 Effects of NMT on tennis-specific and physical performance

First, stroke velocity was one of the tennis-specific outcomes for which the pooled estimates most consistently favored the intervention conditions in the present review. The direction of the intervention effect was consistent across peak serve velocity, mean serve velocity, and groundstroke velocity. This pattern indicates that the favorable direction was observed across different stroke-velocity indicators rather than being confined to a single measurement approach. Unlike previous reviews that used broader constructs such as stroke velocity or maximal serve velocity, the present analysis separated peak serve velocity, mean serve velocity, and groundstroke velocity [45]. Previous reviews have also discussed possible links between serve-velocity changes and kinetic-chain transmission, intermuscular coordination, and neuromuscular recruitment patterns [46–48]. Notably, age subgroup analyses showed a larger pooled effect for stroke velocity in young adults than in youth. However, because these analyses were exploratory and based on chronological age only, this pattern should not be interpreted as evidence that chronological age is an established moderator of training responses.

Compared with stroke velocity, the accuracy-related outcomes showed a more differentiated response pattern. Specifically, the pooled estimate favored the intervention conditions for serve accuracy, whereas no significant pooled effect was observed for shot placement accuracy. This difference suggests that accuracy should not be treated as a unitary construct, because different tests may reflect different levels of tennis-specific control. Because the included studies used different testing protocols and scoring systems, serve accuracy and shot placement accuracy were interpreted separately and cautiously. One possible explanation is that serve accuracy was assessed in a more constrained and repeatable test context than shot placement accuracy. By contrast, shot placement accuracy may involve multiple technical, perceptual, and task-specific factors, including stroke timing, racket-face control, incoming-ball processing, perceptual-decisional processes, and tactical execution. However, these factors were not consistently measured in the included studies, so this interpretation should be regarded as a hypothesis rather than a mechanism directly tested in the included studies. Previous reviews have provided limited or inconsistent conclusions regarding accuracy-related outcomes [5,12,45]. The more refined accuracy classification used in the present study further suggests that different accuracy outcomes may not respond uniformly to training stimuli. Given the small number of included studies and the very low certainty of evidence, the accuracy-related findings should be interpreted as preliminary and should not be taken as evidence of a uniform effect of NMT on tennis-specific accuracy.

For physical performance, the findings for sprint performance have potential tennis-specific relevance. The present study suggests that the improvement signal was more consistent for the 10 m sprint than for the 20 m sprint, which aligns well with the movement demands of tennis, where athletes frequently perform short-distance starts, accelerations, decelerations, and reaccelerations [1,2]. Compared with longer linear sprint distances, 10 m may better reflect first-step explosiveness and short-distance acceleration capacity in tennis and may therefore offer greater sport-specific relevance. However, because the subgroup difference between the 10 m and 20 m sprint did not reach statistical significance, this interpretation should remain cautious. Notably, the 20 m sprint was not significant in the SMD-based analysis but showed a small yet significant improvement in the MD-based analysis. This pattern suggests that the training effect for the 20 m sprint may be less stable across effect-size metrics. Because SMD is more sensitive to within-study variability and testing differences, whereas MD preserves the original unit of seconds, some inconsistency between these two metrics is not unexpected.

The pooled results for CMJ and agility favored the intervention conditions; however, these findings require particular caution because the certainty of evidence was very low. CMJ and agility were included as performance outcomes rather than direct measures of underlying neuromuscular mechanisms. Cross-sectional evidence from youth basketball and football athletes suggests that speed, agility, and jump performance are associated with several anthropometric and physical-performance characteristics, whereas overall functional movement quality may not be consistently associated with these outcomes [49,50]. Possible explanations for the favorable pooled estimates include improvements in explosive power, stretch-shortening-cycle function, braking ability, or change-of-direction control [3,45]. However, these potential mechanisms were rarely measured directly in the included studies. Evidence from previous reviews has shown that athletic training produces substantial effects on agility and lower-extremity muscle power [3]. More specifically, plyometric training can also improve sprint performance, lower-extremity muscle power, and agility, which is consistent with the overall direction of the present findings [45]. Furthermore, age subgroup analyses showed effects favoring the intervention conditions for agility in both youth and young adult male players, with no significant between-group difference. By contrast, stroke velocity showed a more pronounced age-related pattern. However, these age-related findings should be interpreted cautiously because chronological age may not adequately capture biological maturation, pubertal status, training history, or competitive level, and these variables were rarely reported in the included studies. This pattern may indicate that age-related differences are not uniform across outcomes, but this interpretation remains exploratory [51]. It should also be noted that both CMJ and agility showed potential small-study effects; accordingly, the magnitude of these effects should be interpreted with caution, and their evidence quality and overall robustness are discussed further in Section 4.4. Therefore, the findings for CMJ and agility should be viewed as tentative rather than confirmatory evidence of training effectiveness.

### 4.3 Comparison with previous reviews and the added value of the present study

Compared with previous reviews, the present study included only male tennis players, thereby avoiding the direct pooling of male and female samples and reducing potential confounding arising from sex-based differences in training responses [5,45]. It separately analyzed 10 m and 20 m sprint performance, peak serve velocity, mean serve velocity, groundstroke velocity, serve accuracy, and shot placement accuracy [3,45]. By contrast, the review by Zhou et al. (2025) included broader participant samples and wider outcome categories [5]. Age-related differences were also explored where data permitted.

Taken together, the contribution of this review lies in organizing a heterogeneous body of interventions within a common review framework and synthesizing the evidence using a more refined outcome structure. This approach helps identify which outcome domains show relatively consistent signals and which remain uncertain. However, the certainty and generalizability of the findings remain limited by the broad age range, intervention heterogeneity, and overall evidence quality.

### 4.4 Evidence quality

Although the direction of most pooled effects was relatively consistent and sensitivity analyses generally supported the direction of the main findings, GRADE assessments indicated that the certainty of evidence was low or very low for all main outcomes. This pattern is broadly consistent with the conclusions of previous reviews and suggests that limited evidence quality remains a common methodological challenge in this field [5,45,52]. Therefore, the present findings are better interpreted as cautious supportive signals that are relatively consistent in direction rather than as conclusions firmly established by high-certainty evidence.

This cautious interpretation is also warranted because the included studies differed in intervention content, training duration and frequency, participant characteristics, and performance-test protocols. Consequently, the findings should not be interpreted as evidence for any single training modality, testing approach, or specific neuromuscular mechanism. In addition, the findings for CMJ and agility require particular caution. Although the direction of effect remained unchanged after trim-and-fill analysis, the magnitude of the effects was attenuated, suggesting that small-study effects or publication bias cannot be completely excluded. Therefore, the estimated effects for CMJ and agility may have been inflated and should be interpreted cautiously. By contrast, the findings for stroke velocity and 10 m sprint were directionally more stable, although the certainty of evidence remained low. Because short-distance acceleration is relevant to tennis movement demands, the 10 m sprint finding may have sport-specific relevance, but this interpretation should remain cautious [1,2,53]. Overall, the interpretation of all outcomes should remain aligned with the low or very low GRADE certainty.

### 4.5 Limitations

This study has several limitations. First, the evidence included both RCTs and non-randomized studies, and continuous outcomes were preferentially pooled using post-intervention values because change scores were not consistently reported. Although baseline comparability was assessed for the non-randomized studies and sensitivity analyses excluding these studies did not materially alter the direction of the pooled effects, the serve accuracy estimate was no longer statistically significant, and residual baseline imbalance cannot be ruled out. Second, relatively few studies reported accuracy-related outcomes, particularly shot placement accuracy, limiting more detailed subgroup comparisons and bias assessments. Third, studies varied substantially in intervention duration, training frequency, loading parameters, training content, and testing procedures. Although interventions were classified according to their dominant training component, intervention-type subgroup analyses were not conducted because several categories contained too few studies or effect sizes to support reliable comparisons. Therefore, the pooled estimates represent average effects across a heterogeneous group of interventions and cannot establish the effectiveness of any specific training modality or determine whether the pooled findings were driven by any particular modality. Fourth, the broad age range and limited reporting of Tanner stage, biological maturation, training age, and competitive level constrained interpretation of the pooled estimates and exploratory age-subgroup findings. Fifth, the available evidence mainly reflected short-term effects, and follow-up data were lacking. Consequently, the durability of the observed effects remains unclear. Sixth, because only English-language full-text articles were included, language bias cannot be ruled out.

### 4.6 Practical implications

From a practical perspective, the present findings suggest that NMT may warrant cautious consideration as one possible component of training programs for male tennis players, particularly when targeting stroke velocity and short-distance sprint performance. However, it would be inappropriate to assume that NMT can comprehensively improve all accuracy-related aspects of tennis-specific performance. More specifically, the favorable signal for serve accuracy should be interpreted cautiously, whereas the evidence for shot placement accuracy remains insufficient to support specific training implications. Because the age subgroup analyses were exploratory and based on chronological age only, the observed age-related pattern for stroke velocity should not be used to support age-specific training recommendations. Overall, the findings suggest potential relevance for selected physical qualities and tennis-specific outcomes in male tennis players, particularly youth and young adults, but do not support NMT as an evidence-confirmed standalone intervention capable of addressing all technical and performance challenges. Given the low or very low certainty of evidence for most outcomes, these implications should be regarded as tentative and should not be interpreted as strong practical recommendations.

## 5. Conclusion

In summary, when the included interventions were considered collectively, the pooled estimates favored the intervention conditions for stroke velocity and short-distance sprint performance in youth and young adult male tennis players, but the certainty of evidence for these outcomes was low. Pooled estimates for CMJ, agility time, and serve accuracy also favored the intervention conditions; however, the certainty of evidence for these outcomes was very low, and accuracy-related findings remained inconsistent. These pooled findings do not establish the effectiveness of any specific training modality. Given the predominantly low or very low certainty of evidence, these findings should be interpreted cautiously and confirmed in well-designed randomized trials.

## Supporting information

S1 Table Database search strategies. Full search strategies used for PubMed, Web of Science, Cochrane Library, Embase, and Scopus.
(DOCX)

S2 Table Baseline comparability of non-randomized studies. Baseline comparisons between intervention and control groups in the two non-randomized controlled studies.
(DOCX)

S3 Table Classification of neuromuscular training interventions. Study-level classification of neuromuscular training interventions according to dominant training component and reported intervention content.
(DOCX)

S4 Table Sensitivity analyses of pooled effects. Sensitivity analyses comparing random-effects models, fixed-effect models, exclusion of non-randomized studies, and targeted analysis excluding the CMJ comparison with potential baseline imbalance.
(DOCX)

S1 Fig Forest plot of pooled effects on performance outcomes. Forest plots showing the pooled effects of neuromuscular training on stroke velocity, sprint performance, countermovement jump, agility time, shot placement accuracy, and serve accuracy.
(TIFF)

S1 Checklist PRISMA 2020 checklist. Completed PRISMA 2020 checklist for this systematic review and meta-analysis.
(DOCX)
