# Supplementary material for: Effects of neuromuscular training on physical and tennis-specific performance in youth and young adult male tennis players: A systematic review and meta-analysis
Source: PLoS One. 2026 Aug 5;21(8):e0355297. doi: 10.1371/journal.pone.0355297 (PMC13440869; doi:10.1371/journal.pone.0355297)
Supplement: Supplementary file 1 [file pone.0355297.s001.docx]

**Supplementary Table S1. Database search strategies**

| **Database** | **Main search strategy** |
| --- | --- |
| **PubMed** | ("Tennis"[Mesh] OR tennis[tiab] OR "tennis player*"[tiab])  AND  ("neuromuscular training"[tiab] OR plyometr*[tiab] OR "resistance training"[tiab] OR "strength training"[tiab] OR "isometric training"[tiab] OR "core training"[tiab] OR "core stability"[tiab] OR "balance training"[tiab] OR "coordination training"[tiab] OR "sprint training"[tiab] OR "agility training"[tiab] OR "medicine ball"[tiab] OR "elastic resistance"[tiab] OR "flywheel training"[tiab] OR "functional training"[tiab]) |
| **Web of Science** | TS=(tennis OR "tennis player*")  AND  TS=("neuromuscular training" OR plyometr* OR "resistance training" OR "strength training" OR "isometric training" OR "core training" OR "core stability" OR "balance training" OR "coordination training" OR "sprint training" OR "agility training" OR "medicine ball" OR "elastic resistance" OR "flywheel training" OR "functional training") |
| **Cochrane Library** | #1[mh "Tennis"]  #2 (tennis OR "tennis player*"):ti,ab,kw  #3 #1 OR #2  #4 ("neuromuscular training" OR plyometr* OR "resistance training" OR "strength training" OR "isometric training" OR "core training" OR "core stability" OR "balance training" OR "coordination training" OR "sprint training" OR "agility training" OR "medicine ball" OR "elastic resistance" OR "flywheel training" OR "functional training"):ti,ab,kw  #5 #3 AND #4 |
| **Embase** | ('tennis'/exp OR tennis:ti,ab,kw OR 'tennis player*':ti,ab,kw)  AND  ('neuromuscular training':ti,ab,kw OR plyometr*:ti,ab,kw OR 'resistance training':ti,ab,kw OR 'strength training':ti,ab,kw OR 'isometric training':ti,ab,kw OR 'core training':ti,ab,kw OR 'core stability':ti,ab,kw OR 'balance training':ti,ab,kw OR 'coordination training':ti,ab,kw OR 'sprint training':ti,ab,kw OR 'agility training':ti,ab,kw OR 'medicine ball':ti,ab,kw OR 'elastic resistance':ti,ab,kw OR 'flywheel training':ti,ab,kw OR 'functional training':ti,ab,kw) |
| **Scopus** | TITLE-ABS-KEY(tennis OR "tennis player*")  AND  TITLE-ABS-KEY("neuromuscular training" OR plyometr* OR "resistance training" OR "strength training" OR "isometric training" OR "core training" OR "core stability" OR "balance training" OR "coordination training" OR "sprint training" OR "agility training" OR "medicine ball" OR "elastic resistance" OR "flywheel training" OR "functional training") |

**Note.** Search strategies were adapted to the syntax and indexing system of each database. No sex restriction was applied during the database search; eligibility by sex was determined during study screening. Searches were conducted from database inception to 1 January 2026.
