# Supplementary material for: Effects of neuromuscular training on physical and tennis-specific performance in youth and young adult male tennis players: A systematic review and meta-analysis
Source: PLoS One. 2026 Aug 5;21(8):e0355297. doi: 10.1371/journal.pone.0355297 (PMC13440869; doi:10.1371/journal.pone.0355297)
Supplement: Supplementary file 2 [file pone.0355297.s002.docx]

**Supplementary Table S2. Baseline comparability of non-randomized studies**

| **Study** | **Design** | **Outcome** | **Intervention baseline M ± SD** | **Control baseline M ± SD** | **Baseline SMD** | **Baseline comparability** | **Action taken** |
| --- | --- | --- | --- | --- | --- | --- | --- |
| Fernandez-Fernandez et al. (2015) | Non-RCT | 10 m sprint, s | 1.84 ± 0.10 | 1.86 ± 0.10 | -0.20 | Comparable / small difference | Retained |
| Fernandez-Fernandez et al. (2015) | Non-RCT | 20 m sprint, s | 2.99 ± 0.10 | 3.01 ± 0.10 | -0.20 | Comparable / small difference | Retained |
| Fernandez-Fernandez et al. (2015) | Non-RCT | CMJ, cm | 41.70 ± 2.10 | 40.50 ± 1.00 | 0.73 | Potential baseline imbalance | Sensitivity analysis conducted |
| Fernandez-Fernandez et al. (2016) | Non-RCT | Serve velocity, km/h | 138.60 ± 8.20 | 140.10 ± 9.40 | -0.17 | Comparable | Retained |
| Fernandez-Fernandez et al. (2016) | Non-RCT | Serve accuracy, points | 11.40 ± 2.50 | 11.00 ± 2.00 | 0.18 | Comparable | Retained |
| Fernandez-Fernandez et al. (2016) | Non-RCT | 10 m sprint, s | 2.01 ± 0.10 | 2.00 ± 0.10 | 0.10 | Comparable | Retained |
| Fernandez-Fernandez et al. (2016) | Non-RCT | 20 m sprint, s | 3.54 ± 0.20 | 3.54 ± 0.20 | 0.00 | Comparable | Retained |
| Fernandez-Fernandez et al. (2016) | Non-RCT | CMJ, cm | 30.10 ± 4.30 | 30.30 ± 4.30 | -0.05 | Comparable | Retained |
| Fernandez-Fernandez et al. (2016) | Non-RCT | Agility / 505 test, s | 2.95 ± 0.20 | 2.93 ± 0.10 | 0.13 | Comparable | Retained |

**Note.** Baseline SMD was calculated as the standardized difference between intervention and control groups at baseline. Absolute values <0.20 were considered negligible, 0.20-0.50 small, and >0.50 potentially important. For time-based outcomes, positive or negative signs indicate direction only and were not used to judge comparability. CMJ, countermovement jump; RCT, randomized controlled trial; SMD, standardized mean difference.
