# Supplementary material for: Effects of neuromuscular training on physical and tennis-specific performance in youth and young adult male tennis players: A systematic review and meta-analysis
Source: PLoS One. 2026 Aug 5;21(8):e0355297. doi: 10.1371/journal.pone.0355297 (PMC13440869; doi:10.1371/journal.pone.0355297)
Supplement: Supplementary file 3 [file pone.0355297.s003.docx]

**Supplementary Table S3. Classification of neuromuscular training interventions**

| **Study** | **Intervention content as reported** | **Dominant intervention category** | **Classification rationale** |
| --- | --- | --- | --- |
| Baiget et al. (2022) | Joint-specific isometric strength training | Resistance/strength-based | Classified according to the use of joint-specific isometric strength exercises. |
| Behringer et al. (2013) | Traditional resistance training and plyometric training | Resistance- and plyometric-based | Classified according to the inclusion of resistance and plyometric training conditions. |
| Canós et al. (2022) | Machine-based neuromuscular training and flywheel neuromuscular training | Resistance/flywheel-based | Classified according to machine-based and flywheel resistance-oriented training. |
| Fernandez-Fernandez et al. (2013) | Core strength, elastic resistance, and medicine ball exercises | Multicomponent | Classified as multicomponent because the intervention combined core, resistance, and medicine-ball exercises. |
| Fernandez-Fernandez et al. (2015) | Combined explosive strength and repeated sprint training | Multicomponent | Classified as multicomponent because the intervention combined explosive strength and repeated sprint training. |
| Fernandez-Fernandez et al. (2016) | Upper- and lower-body integrated plyometric training | Plyometric-based | Classified according to the dominant plyometric training component. |
| Gamlath and Thotawaththa (2023) | Lower-extremity plyometric activities | Plyometric-based | Classified according to the dominant lower-limb plyometric training component. |
| Genevois et al. (2013) | Handled medicine ball and overweight racket training | Loaded power-based | Classified according to loaded upper-limb and stroke-related power training. |
| Kong and Xu (2023) | Progressive medicine ball strength training | Resistance/power-based | Classified according to progressive medicine-ball strength and power-oriented training. |
| Mainer-Pardos et al. (2024) | Speed, strength, throws, agility, jumps, and coordination training | Multicomponent | Classified as multicomponent because the intervention combined several neuromuscular training elements. |
| Paul et al. (2011) | Sport-specific agility and footwork drills | Agility/footwork-based | Classified according to the dominant agility and footwork training component. |
| Rathore (2016) | Plyometric training and traditional resistance training | Resistance- and plyometric-based | Classified according to the inclusion of both plyometric and resistance training approaches. |
| Salonikidis and Zafeiridis (2008) | Plyometric training, tennis-specific drills, and combined training | Multicomponent | Classified as multicomponent because the intervention included plyometric, tennis-specific, and combined training conditions. |
| Sinkovic et al. (2023) | Lower-limb plyometric training | Plyometric-based | Classified according to the dominant lower-limb plyometric training component. |
| Wang and Xu (2025) | Upper-limb plyometric training | Plyometric-based | Classified according to the dominant upper-limb plyometric training component. |
| Yildiz et al. (2019) | Functional training | Functional/multicomponent | Classified as multicomponent because functional training may include combined strength, coordination, and movement-control elements. |
| Ölçücü et al. (2013) | Progressive plyometric training | Plyometric-based | Classified according to the dominant plyometric training component. |
| Ziagkas et al. (2019) | Plyometric training integrated into tennis practice | Plyometric-based | Classified according to the dominant plyometric training component integrated into tennis practice. |

**Note.** NMT, neuromuscular training. Studies were descriptively classified according to the dominant training component and the intervention content reported in the included studies. These categories were used to characterize clinical heterogeneity across interventions and do not imply that the included modalities are biomechanically equivalent, interchangeable, or expected to produce identical training effects. This classification was not used as a basis for formal subgroup analysis because several categories contained too few studies or effect sizes to support reliable comparisons.
