# Supplementary material for: Effects of neuromuscular training on physical and tennis-specific performance in youth and young adult male tennis players: A systematic review and meta-analysis
Source: PLoS One. 2026 Aug 5;21(8):e0355297. doi: 10.1371/journal.pone.0355297 (PMC13440869; doi:10.1371/journal.pone.0355297)
Supplement: Supplementary file 4 [file pone.0355297.s004.docx]

**Supplementary Table S4. Sensitivity analyses of pooled effects**

| **Outcome** | **Primary random-effects model, g [95% CI]; p; I²** | **Fixed-effect model, g [95% CI]; p; I²** | **Excluding non-randomized studies, g [95% CI]; p; I²** | **Additional targeted analysis** | **Interpretation** |
| --- | --- | --- | --- | --- | --- |
| Stroke velocity | 0.95 [0.68, 1.22]; p < 0.001; I² = 23% | 0.97 [0.75, 1.20]; p < 0.001; I² = 23% | 1.03 [0.75, 1.31]; p < 0.001; I² = 15% | Not applicable | Direction and statistical significance unchanged |
| Sprint performance | -0.58 [-0.87, -0.28]; p = 0.0001; I² = 19% | -0.56 [-0.82, -0.31]; p < 0.0001; I² = 19% | -0.57 [-0.95, -0.18]; p = 0.004; I² = 35% | Not applicable | Direction and statistical significance unchanged |
| Countermovement jump (CMJ) | 0.73 [0.34, 1.13]; p = 0.0003; I² = 38% | 0.61 [0.32, 0.90]; p < 0.0001; I² = 38% | 0.74 [0.29, 1.19]; p = 0.001; I² = 30% | Excluding the CMJ comparison with potential baseline imbalance: 0.63 [0.25, 1.01]; p = 0.001; I² = 30% | Direction and statistical significance unchanged |
| Agility time | -0.89 [-1.22, -0.56]; p < 0.001; I² = 32% | -0.82 [-1.08, -0.56]; p < 0.001; I² = 32% | -0.98 [-1.32, -0.63]; p < 0.001; I² = 23% | Not applicable | Direction and statistical significance unchanged |
| Shot placement accuracy | -0.41 [-0.92, 0.11]; p = 0.12; I² = 1% | -0.41 [-0.92, 0.10]; p = 0.12; I² = 1% | -0.41 [-0.92, 0.11]; p = 0.12; I² = 1% | Not applicable | Remained non-significant |
| Serve accuracy | 0.47 [0.11, 0.84]; p = 0.01; I² = 13% | 0.47 [0.13, 0.81]; p = 0.007; I² = 13% | 0.51 [-0.03, 1.04]; p = 0.06; I² = 34% | Not applicable | Direction unchanged, but no longer statistically significant after excluding non-randomized studies |

Note. Values are Hedges' g with 95% confidence intervals. Negative values for sprint performance, agility time, and shot placement accuracy indicate favorable effects when lower scores reflect better performance. The analysis excluding non-randomized studies was conducted to examine whether pooled estimates were influenced by non-randomized evidence. CMJ, countermovement jump; CI, confidence interval; I², heterogeneity statistic.
